# Stability of short-term boldness personality under nutritional and disturbance stress in a social spider

**DOI:** 10.1186/s12983-026-00612-7

**Published:** 2026-05-01

**Authors:** Bharat Parthasarathy, Klementien Friedrich, Jutta M. Schneider

**Affiliations:** Institute of Animal Cell and Systems Biology (IZS), University of Hamburg, Martin-Luther-King Platz 3, 20146 Hamburg, Germany

**Keywords:** Repeatability of behaviour, *Stegodyphus sarasinorum*, Hunger state, Starvation, Huddle, Latency to move, Animal activity tests

## Abstract

**Background:**

Consistent among-individual differences in behaviours, also known as animal personalities, are ubiquitous across the animal kingdom. At the same time, the expression of these behaviours may remain context- and time- dependent. Social spiders lack morphological castes and apparent dominant hierarchies, but show consistent among-individual differences in behaviours such as boldness and aggression. Previous studies have shown that these personality traits are not associated with task participation and that personality repeatability weakens over longer durations. In this study, we tested whether short-term boldness was stable across intrinsic (nutritional state) and extrinsic (disturbance) contexts in the Indian social spider, *Stegodyphus sarasinorum*. We subjected individuals to feeding or starvation and repeated disturbance or no disturbance treatments, and measured boldness over three consecutive days.

**Results:**

We found that neither variation in nutritional state nor disturbance influenced the mean boldness scores or their repeatability. Our results show that boldness is robust to short-term changes in hunger and disturbance.

**Conclusion:**

The ecological and social functions of the boldness personality trait remain unresolved in social spiders and further studies are warranted to understand how exactly among-individual variation in boldness influences colony productivity and demographic outcomes.

**Supplementary Information:**

The online version contains supplementary material available at 10.1186/s12983-026-00612-7.

## Background

Across the animal kingdom, individuals within populations differ consistently in behaviours such as boldness, aggression, exploration, and sociability [1–3]. Such consistent among-individual differences in behaviours, also known as animal personalities, can persist across time and/or contexts [4–6], and have been well documented across a wide range of taxa, including invertebrates [7–9]. Within the framework of animal personality, behavioural expression refers to the behaviour of an individual in a given context, whereas consistency refers to the rank-order differences in behaviour among individuals across time and/or contexts [10, 11].

Although behavioural expression can be consistent, it can also vary with changes in internal state and external environmental conditions [12, 13]. One important axis along which behavioural expression shows plasticity is an animal’s nutritional condition. Hunger and nutritional states are known to influence foraging and risk-taking behaviours in animals [14–16]. Similarly, disturbance or exposure to threat may change the behavioural expression of individuals [17]. Such context-dependent changes reflect behavioural plasticity, defined as within-individual changes in behaviour across contexts. If such contexts exert similar effects among individuals, they may shift mean behavioural expression at the population level. However, if individuals differ in the extent to which their behaviour changes across contexts, they may also alter the consistency of among-individual differences [12, 13] and hence repeatability (quantified as the proportion of total behavioural variance attributable to differences among individuals [18]). On the other hand, if personalities are robust to changes in state or context, then both mean behavioural expression and repeatability should persist despite such perturbations.

Social spiders are a powerful system to address these questions. Unlike many social insects such as bees, ants and wasps, social spiders lack morphological castes and apparent dominance hierarchies [19]. However, individuals show consistent among-individual differences in behavioural traits such as boldness and aggression, although the repeatability of these traits weakens over the long term [20, 21]. Moreover, in the Indian social spider *Stegodyphus sarasinorum*, these personality traits were not associated with task participation [22]. However, it remains unclear whether short-term behavioural expressions are influenced by transient states such as hunger or recent exposure to disturbance. Addressing this knowledge gap is important not only for interpreting the functional significance of personality, but also for understanding the mechanisms that govern the maintenance of among-individual differences in behaviours in a system lacking clear social hierarchies.

In this study, we followed a split-brood experimental design to test whether short-term boldness in *S. sarasinorum* is stable across intrinsic and extrinsic contexts. By experimentally manipulating nutritional state (fed versus starved) and disturbance (repeated abdominal prodding versus no prodding), we measured boldness over three consecutive days. We asked whether these treatments affected mean boldness expression (i.e., mean boldness scores) and repeatability in boldness. If boldness is state dependent and plastic in response to disturbance, we expected that the two treatments would influence mean behavioural expression and reduce repeatability. Alternatively, if boldness is a stable short-term personality trait, we predicted that among-individual differences would persist and repeatability would be maintained across the treatments.

## Methods

**Spider Collection**,** Housing and Construction of Experimental Colonies**: We collected *Stegodyphus sarasinorum*, also known as the Indian social spider, along a 161 km transect from Madurai to Tirunelveli in Tamil Nadu, Southern India in December 2021. We transported the spiders to the laboratory at the University of Hamburg, Germany after obtaining permits from the National Biodiversity Authority of India (permit no: NBA/Tech Appl/9/Form B-152/20/20–21/787). Colonies were maintained in a climate chamber at 26 °C, 60% relative humidity and 12 h light: dark cycle. We fed the colonies with blow flies (*Calliphoridae*) twice a week and sprinkled water daily. We created 18 experimental colonies, each comprising 10 randomly selected spiders from 9 different source (parent) colonies, such that individuals from each source colony were distributed across two experimental colonies. Each spider was uniquely marked on their abdomens with non-toxic water colours. Of these eighteen experimental colonies, we fed nine colonies to satiation with blow flies while the remaining nine colonies were starved for 7 days. Based on our previous experiments, we knew that 7 days of starvation would be sufficient to stress the spiders without causing death by starvation [22]. We subjected five spiders from each experimental colony to stress (disturbed spiders) by prodding their abdomens with a paint brush three times. After prodding each time, we allowed spiders to remain still before prodding their abdomens again. Subsequently, we allowed the spiders to settle for 1 min after which we performed the boldness assays as described below. We did not stress the remaining five spiders (undisturbed spiders) and they were subjected to boldness assays after 1 min of acclimatization. Therefore, we had a 2 × 2 fully factorial (split-brood) design, with food treatment applied at the experimental colony level (fed versus starved) and disturbance applied within experimental colonies (disturbed versus undisturbed), generating four treatment combinations: (1) stressed and fed, (2) stressed and starved, (3) undisturbed and fed, and (4) undisturbed and starved.

**Boldness assay**: Boldness assays were performed exactly as described by Parthasarathy et al. [22]. We performed 3 boldness assays over 3 days (1 assay/day). 11 out of 180 spiders died during various stages of the experiments and therefore we had a total of 516 observations across colonies for the three trials. We isolated spiders individually into plastic Petri dishes (diameter: 3 cm; depth: 1.5 cm) for 1 h. Subsequently, we placed the spiders inside a rectangular plastic dish (14.5 × 11.2 × 5.2 cm) and allowed them to acclimatize for 1 min. We delivered a rapid puff of air on the spider using a 5 ml plastic syringe. Spiders reacted to the air puff by withdrawing their legs under their body (huddling). We noted the time (*s*) it took for the spider to resume its movement. Spiders that did not react to the air puff were given a score of 0 s (47/516 observations) and we stopped the assays if a spider did not resume its movement after 1200 s (49/516 observations). Spiders that took longer latencies to resume movement were deemed ‘shy’ whereas spiders that moved after short latencies were deemed ‘bold’.

### Statistical analyses

We performed all our analyses in R. We included the partial data of dead spiders in our analyses. As 96 out of 516 observations (18.6%) were either 0 *s* or 1200 *s*, we performed [0,1] beta inflated generalized additive models for location, scale and shape using the ‘GAMLSS’ package in R [23] to determine the effect of food and disturbance on boldness scores. Boldness scores were converted into proportions by dividing each observation by the maximum boldness score of 1200 *s*. The boldness score (in proportion) was the dependent variable. The independent variables were trial numbers, food status (whether fed or starved) and stress status (whether disturbed or undisturbed). To account for the hierarchical structure of the data, we included random intercepts for parental colony, experimental colony nested within parental colony, and individual identity nested within experimental colony, thereby accounting for shared genetic background, shared environmental conditions, and repeated measures of individuals. An interaction term between food and stress status was included in the model. Since the interaction between food and disturbance was not significant, we report the model refitted without the interaction term to focus on the main effects. We diagnosed the model by inspecting the randomized quantile residuals, including Q-Q plots, worm plots (detrended Q-Q plots) and residual versus fitted values. Quantile residuals were approximately normally distributed and centred around zero, with no apparent systematic patterns in residuals across fitted values. Moreover, the deviations in the worm plots were small and largely within the simulated confidence envelopes, thus indicating no major violations of model assumptions. We estimated repeatability of boldness among the treatment groups using the ‘MCMCglmm’ package in R [24]. We converted the boldness scores into an ordered variable (coded as 1 for boldness scores between 0 and 100 s; 2 for 101 and 200, and so on) and used this ordered variable as the response variable and spider identity as the random effect, with separate individual-level variance components estimated for each food × disturbance treatment combination. We fixed the residual variance on the latent scale to 1 (as required by threshold models). We used parameter expanded priors for random effects and weakly informative priors for the residual variance. Chains were run for 1,000,000 iterations, with a thinning interval of 1000. We checked for model convergence and chain mixing by examining trace plots, effective sample sizes, and Heidelberger–Welch tests.

### AI Usage

This manuscript employed AI assistance (ChatGPT 5.2 from OpenAI) for improving the clarity of the language. All scientific design, validation, interpretation and manuscript writing were performed by the authors.

## Results

### Food treatment and disturbance have no effect on short-term boldness

Our generalized additive model for location, scale and shape (GAMLSS) showed that neither food treatment (fed versus starved) nor disturbance (prodded versus undisturbed) influenced short-term (3 days) boldness in *Stegodyphus sarasinorum* (Table 1). This shows that mean expression in boldness is not influenced by variation in nutritional state and disturbance, at least in the short term. Trials had a significant effect on boldness (Table 1). As trials progressed, spiders became progressively shyer, as they took longer to resume movement across successive trials.

**Table 1:** The effects of food, disturbance, and trial on boldness: The estimates from the GAMLSS model are in logit scale

Effect	Estimate	SE	t value
Intercept	−1.081	0.123	−8.77^*^
Food (fed)	0.026	0.080	0.33
Disturbance (disturbed)	0.064	0.080	0.81
Trial	0.099	0.049	2.01^*^

^*^ represents *P* < 0.05. Food and disturbance have no effect on boldness scores in *Stegodyphus sarasinorum*

### Repeatability of boldness did not differ among treatment groups

**Fig. 1:**
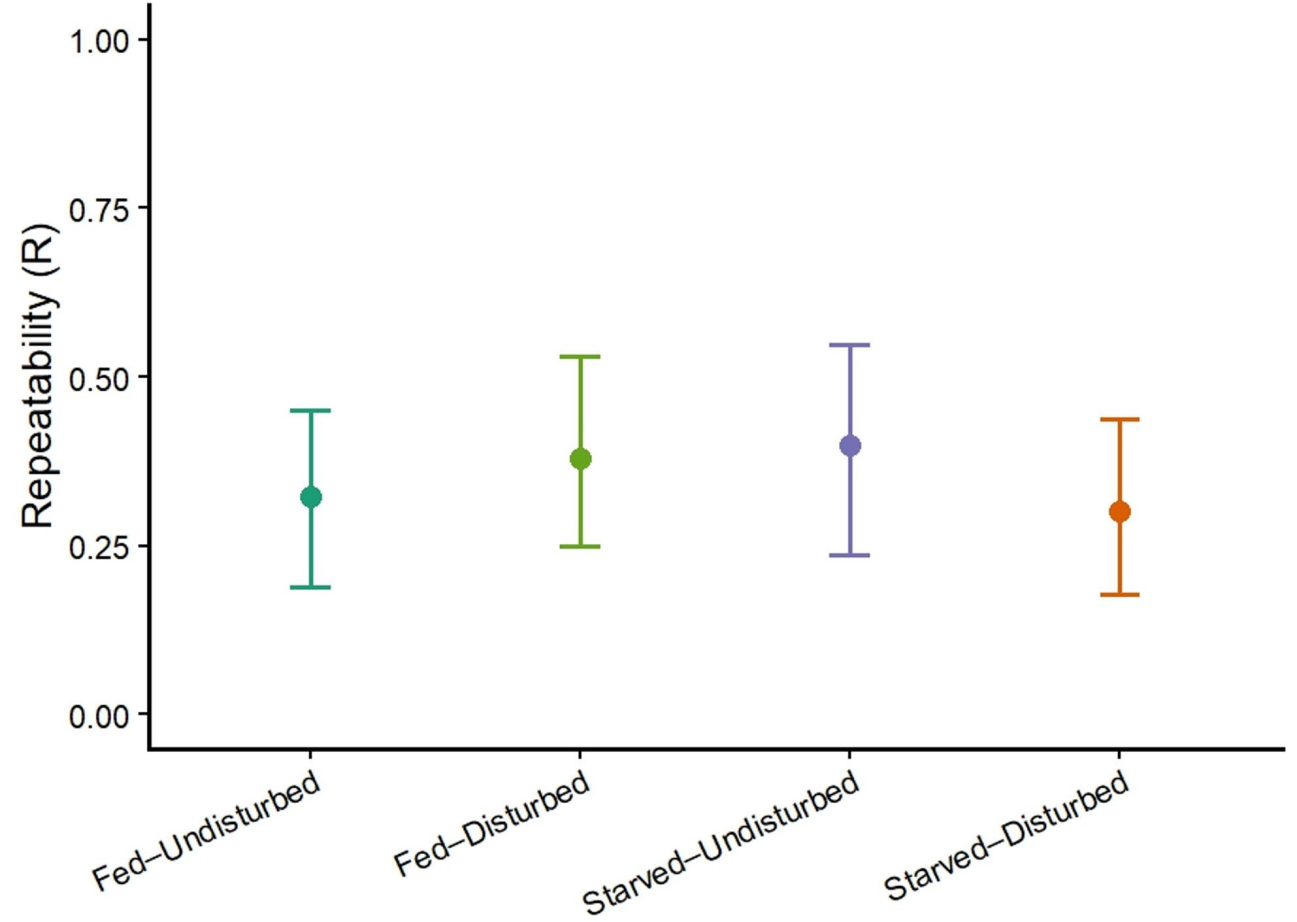
Treatment-specific repeatability of boldness in *S. sarasinorum*. Points show posterior means of repeatability (R) for boldness, estimated from ordinal threshold model. Error bars indicate 95% highest posterior density (HPD) intervals. Repeatability did not differ among food (fed vs. starved) or disturbance (disturbed vs. undisturbed) treatments

Our threshold MCMCglmm model showed that boldness scores had similar repeatability estimates across fed & disturbed, fed & undisturbed, starved & disturbed and starved & undisturbed treatment groups (Fig. 1; Table 2). Posterior contrasts provided no credible evidence that variation in nutritional status and disturbance altered the repeatability of the boldness personality trait in *S. sarasinorum*.

**Table 2:** Treatment-specific repeatability of boldness and posterior contrasts: The MCMCglmm model shows that repeatability in boldness scores is similar across the four treatment groups in *S. sarasinorum*

Treatment	Repeatability (*R*)	95% HPD interval
Fed & Undisturbed	0.323	0.189–0.450
Fed & Disturbed	0.379	0.250–0.531
Starved & Undisturbed	0.399	0.237–0.547
Starved & Disturbed	0.302	0.178–0.437

Contrast	ΔR	95% HPD interval
Starved – Fed (undisturbed)	0.076	−0.141–0.266
Disturbed – Undisturbed (starved)	−0.097	−0.291–0.103
Disturbed – Undisturbed (fed)	0.056	−0.137–0.251

Posterior contrasts show no difference in repeatability among the treatment groups

## Discussion

In this study, we tested whether variation in nutritional state and disturbance influenced boldness expression in the Indian social spider, *Stegodyphus sarasinorum*. Neither food deprivation nor repeated disturbance by abdomen prodding altered mean boldness scores or repeatability of boldness over a three-day period. Our results show that short-term boldness in this species is not an artefact of transient variation in hunger levels or threat exposure. However, boldness scores increased across trials, indicating that spiders became progressively shyer with repeated testing. Previous studies in social spiders have shown that personalities were not associated with task participation of individuals [22] and that they were unstable over longer durations commensurate with the lifespan of the species [20]. At the same time, among-individual differences in behaviours over the short term were stable [21]. Our results extend these findings and suggest that short-term personalities in *S. sarasinorum* are robust to commonly invoked sources of behavioural plasticity such as nutritional state and disturbance. Finally, our results are consistent with the hypothesis that personalities in social spiders may be linked to a relatively slow-changing physiological states rather than short-term energetic condition [9].

Prodding of the abdomen in spiders elicits aggressive (e.g. raising legs) or docile (e.g. running away) defensive responses against invertebrate predators [21, 25–27]. This makes abdomen prodding a standardised disturbance assay. A previous study in *S. sarasinorum* showed that the association between boldness and aggression, also called a behavioural syndrome, persisted only when personalities were observed over longer durations, but not over the short-term [28]. In that study, individuals that responded more aggressively to abdominal prodding also tended to be bolder, yet the assays for aggression and boldness were separated by one hour or more. As a result, it remained unclear whether the observed association reflected a stable behavioural syndrome or whether disturbance itself directly influenced subsequent boldness expression. In this study, we explicitly tested this by repeatedly prodding the abdomen of individuals and measuring boldness after a short acclimation period of one minute. As disturbance did not alter spiders’ huddling latency, our results suggest that short-term expression of boldness is not simply a transient consequence of recent disturbance, but instead reflects a more persistent behavioural tendency.

The boldness personality trait, measured as the spiders’ latency to resume movement following huddling, has been widely interpreted as an anti-predatory response against avian predators across spider taxa [29]. By huddling, these spiders pretend to be dead, which may deter predators that rely on prey movement. However, the adaptive significance of this response against different predator types remains obscure. As our study shows no association between hunger state or disturbance and boldness expression, boldness likely reflects an anti-predator response that is functionally distinct from behaviours directly linked to variation in energetic condition or recent disturbance.

Although we did not measure metabolic rates of spiders, individuals with higher metabolic rates may show shorter latencies to resume movement following huddling. If boldness is linked to intrinsic differences in metabolic rate, bolder individuals may have higher energetic demands and thus may exhibit more frequent foraging responses [30, 31]. However, a previous study in *S. sarasinorum* showed that hunger state strongly influences attack propensity, whereas boldness scores did not predict attack across hunger states [22]. In the present study, we further show that variation in hunger state does not influence boldness expression. Together, these findings indicate that boldness is likely decoupled from both energetic state and foraging behaviour. Future studies combining direct measurements of metabolic rate with boldness assays would be necessary to test whether any link between physiology and personality exists.

## Conclusion

In conclusion, our findings show that short-term boldness in *S. sarasinorum* is stable across ecologically relevant variation in hunger and disturbance. While documenting such behavioural consistency is an important first step, identifying the physiological and neurobiological mechanisms underlying personality variation remains equally important. Without the underlying mechanistic insight, personality studies have limited power to explain how consistent behavioural differences arise and influence social and ecological outcomes.

## Supplementary Information

Below is the link to the electronic supplementary material.

Supplementary Material 1

## Data Availability

All data generated or analysed during this study are included in this published article [and its supplementary information files].
